# Institutional Notes and News

**Published:** 1911-02-11

**Authors:** 


### Institutional Notes and News.

**The Fire at Ealing.**

The fire at Ealing Hospital last week was a dangerous affair and required the best energies of two brigades to subdue it. No patients were hurt. About 9 p.m. on Wednesday night signs of fire were noticed in the western end of the north block, and the matron, caretaker, and half-dozen nurses did their best to put the flames out. It was soon seen, however, that greater efforts must be made, and the telephone quickly brought the Ealing Brigade, under the direction of Chief Inspector Gravener, who turned out with the hose, escape, and steamer, and who was followed shortly afterwards by the Brentford Brigade. On their arrival the upper part of the west end of the north block was blazing steadily, and the roof was burnt out in spite of all efforts. The fire, however, was cut off from the east block, the children were moved to the south block, and three standpipes were made to play upon the burning building. Smoke and water have left their marks on the buildings, but the other blocks, like the children, escaped unharmed. As a final precaution the firemen remained all night with one standpipe in case the fire should break out anywhere again.

**Oakham Cottage Hospital.**

The Countess of Lonsdale (President) occupied the chair at the seventh annual meeting of the Oakham (Rutland) Cottage Hospital. The report stated that 79 patients had been treated during the year, and that an additional nurse was approved for the six months of the year when the work was heaviest. The income amounted to £474, including a balance in hand, and the expenditure to £458 9s. 7d., leaving a small surplus. We note that £50 has been placed on deposit during the year, which is a healthy sign.

**The New Putney Hospital.**

The inhabitants of Putney and the surrounding districts have not been backward in advancing the scheme for the establishment of a general hospital in that growing neighbourhood; indeed, there has been need of a hospital for many years seeing that the nearest hospital is the West London, which is nearly three miles distant. The number of accidents alone warrants the establishment of some centre of medical relief, but beyond this there are many poor persons who are legitimate objects of a general hospital, and who now have to suffer much hardship owing to the distance they have to travel before they can obtain medical or surgical assistance. By the will of the late Mr. H. G. Chester the hospital is placed in an exceptionally good position to establish what may become one of the leading hospitals of London, for the testator has bequeathed a sum of money which is not only enough to build a modern hospital of fifty beds, but which places at the disposal of the trustees a capital sum that will yield over £1,500 per annum towards the maintenance of the institution. Furthermore this income will increase as time goes on, for there is a further capital sum set aside to pay various annuities, and as these fall in the income that will become payable to the charity will increase. Mr. Chester directed that the hospital was to be a general hospital, and that, failing this, the bequest was to revert to Guy's Hospital; but the committee that has this matter in hand will not fail to see that the provisions of the will are carried out and that the neighbourhood shall benefit by the good intention of the testator. Beyond this the residents in the district have been active, and under the chairmanship of Sir William Lancaster, formerly Mayor of Wandsworth, and the hon. secretaryship of Mr. J. F. Taylor, a sum of £1,500 is already in hand towards the new buildings. The site has been given by Sir William Lancaster, and the actual work of construction already has begun.

